# Pneumococcal serotypes in children, clinical presentation and antimicrobial susceptibility in the PCV13 era

**DOI:** 10.1017/S0950268820002708

**Published:** 2020-11-05

**Authors:** C. Izquierdo, P. Ciruela, S. Hernández, J. J. García-García, C. Esteva, F. Moraga-Llop, A. Díaz-Conradi, J. Martínez-Osorio, A. Solé-Ribalta, M. F. de Sevilla, S. González-Peris, G. Codina, A. M. Planes, S. Uriona, M. Campins, C. Muñoz-Almagro, L. Salleras, A. Domínguez

**Affiliations:** 1 Agència de Salut Pública de Catalunya, Generalitat de Catalunya, Barcelona, Spain; 2 CIBER de Epidemiología y Salud Pública (CIBERESP), Madrid, Spain; 3 Hospital Sant Joan de Déu, Universitat de Barcelona, Esplugues de Llobregat, Barcelona, Spain; 4 Malalties prevenibles amb vacunes, Institut de recerca Sant Joan de Déu, Esplugues de Llobregat, Barcelona, Spain; 5 Hospital Vall d’ Hebron, Universitat Autònoma de Barcelona, Barcelona, Spain; 6 Hospital HM Nens, HM Hospitales, Barcelona, Spain; 7 Departament de Medicina. Universitat Internacional de Catalunya, Barcelona, Spain; 8 Departament de Medicina, Universitat de Barcelona, Barcelona, Spain

**Keywords:** Antimicrobial susceptibility, invasive pneumococcal disease, PCV13 vaccine, penicillin resistance, serotype distribution, *Streptococcus pneumoniae*

## Abstract

The aim was to analyse invasive pneumococcal disease (IPD) serotypes in children aged ⩽17 years according to clinical presentation and antimicrobial susceptibility. We conducted a prospective study (January 2012–June 2016). IPD cases were diagnosed by culture and/or real-time polymerase chain reaction (PCR). Demographic, microbiological and clinical data were analysed. Associations were assessed using the odds ratio (OR) and 95% confidence intervals (CI). Of the 253 cases, 34.4% were aged <2 years, 38.7% 2–4 years and 26.9% 5–17 years. Over 64% were 13-valent pneumococcal conjugate vaccine (PCV13) serotypes. 48% of the cases were diagnosed only by real-time PCR. Serotypes 3 and 1 were associated with complicated pneumonia (*P* < 0.05) and non-PCV13 serotypes with meningitis (OR 7.32, 95% CI 2.33–22.99) and occult bacteraemia (OR 3.6, 95% CI 1.56–8.76). Serotype 19A was more frequent in children aged <2 years and serotypes 3 and 1 in children aged 2–4 years and 5–17 years, respectively. 36.1% of cases were not susceptible to penicillin and 16.4% were also non-susceptible to cefotaxime. Serotypes 14, 24F and 23B were associated with non-susceptibility to penicillin (*P* < 0.05) and serotypes 11, 14 and 19A to cefotaxime (*P* < 0.05). Serotype 19A showed resistance to penicillin (*P* = 0.002). In conclusion, PCV13 serotypes were most frequent in children aged ⩽17 years, mainly serotypes 3, 1 and 19A. Non-PCV13 serotypes were associated with meningitis and occult bacteraemia and PCV13 serotypes with pneumonia. Non-susceptibility to antibiotics of non-PCV13 serotypes should be monitored.

## Introduction

*Streptococcus pneumoniae* infections are associated with substantial morbidity and mortality worldwide. The World Health Organization estimated that approximately 335 000 (240 000–460 000) deaths were due to *S. pneumoniae* in children aged <5 years in 2015 [1].

The epidemiology of the invasive pneumococcal disease (IPD) has changed after the introduction of pneumococcal conjugate vaccines [2]. In countries where the 7-valent pneumococcal conjugate vaccine (PCV7) containing serotypes 4, 6B, 9V, 14, 18C, 19F and 23F was introduced, the global IPD incidence decreased but non-PCV7 serotypes emerged [2–5]. Later, the 10-valent pneumococcal conjugate vaccine (PCV10), which included the additional serotypes 1, 5 and 7F, and 13-valent pneumococcal conjugate vaccine (PCV13) which includes the additional serotypes 3, 6A and 19A [6] were introduced, and a significant decline in some additional serotypes was observed [7].

In Catalonia, Spain, PCV7 was available in 2001, but only children with risk factors were vaccinated for free [8, 9]. Estimated vaccine coverage of around 50% was reached [9]. Between 2005 and 2007, a change in IPD serotypes was found in children aged <2 years compared with the pre-vaccine period, with a decrease in PCV7 serotypes and an increase in non-PCV7 serotypes [10]. PCV13 replaced PCV7 in 2010 and, in July 2016, was included in the official paediatric vaccination calendar [11]. PCV13 covered up to 70% of IPD cases in our reference area. In 2013, the estimated coverage of PCV13 in children aged <2 years was 64% (92.5% fully vaccinated) [12].

Studies worldwide have found that the changing distribution of pneumococcal serotypes has led to changes in the rates of *S. pneumoniae* resistance to antibiotics [13, 14].

The aim of this study was to analyse the distribution of *S. pneumoniae* serotypes according to the clinical presentation of IPD and antimicrobial susceptibility in children aged ⩽17 years in a community with intermediate PCV13 coverage before its introduction in the official paediatric vaccination calendar.

## Methods

### Study design

We conducted a prospective observational study between January 2012 and June 2016 in children aged ⩽17 years with IPD attended in three paediatric hospitals: Hospital Vall d'Hebron of Barcelona, Hospital Sant Joan de Déu of Esplugues, Barcelona and Hospital de Nens of Barcelona. The estimated reference population aged ⩽17 years of the hospitals was 442 761, representing 31.9% of this age group in Catalonia.

IPD was diagnosed through the isolation of *S. pneumoniae* by culture or detection of bacterial DNA by real-time polymerase chain reaction (PCR) in any normally sterile site [15], such as blood, cerebrospinal fluid, pleural fluid or articular fluid, among others. We established the presence of *S. pneumoniae* DNA by amplification of the autolysin (*lytA*) gene and the *wzg* (*cpsA*) gene, according to published assays [16–18]. We included only samples that were positive for the *lytA* and *wzg* genes in real-time PCR.

All clinical samples were analysed by culture and real-time PCR.

The clinical evaluation was made by the paediatricians participating in the study according to established diagnostic criteria [19].

### Data collected

Epidemiological and clinical characteristics, including age, gender, underlying medical condition, clinical presentation, intensive care unit (ICU) admission, clinical outcome and PCV13 vaccination status were collected. The method of diagnosis, the serotype and antimicrobial susceptibility were analysed.

A subject was considered vaccinated if they had received the last dose of PCV13 ⩾15 days before symptom onset.

### Serotype identification and antimicrobial susceptibility

Strains isolated by culture were serotyped using the Quellung reaction or dot blot at the National Centre of Microbiology (Majadahonda, Spain) [20]. When the diagnosis was made only by real-time PCR, serotypes were identified at the Molecular Microbiology Department, Hospital Sant Joan de Déu, in accordance with previously-validated methods [15].

Capsular typing of all culture-negative and real-time PCR-positive samples was made using two methods, depending on the amount of *S. pneumoniae* DNA available. If the amount was low (detection of *lytA* gene DNA and an additional capsular gene of *S. pneumoniae* by real-time PCR with the cycle threshold (CT) of >30 cycles), a previously described, real-time multiplex PCR technique that detects all pneumococcal capsular types and differentiates serotypes 1, 3, 4, 5, 6A/C, 6B/D, 7F/A, 8, 9V/A/N/L, 14, 15B/15C, 18C/18B, 19A, 19F/19B/19C, 23A and 23F was used [16]. If the amount of *S. pneumoniae* DNA was high (PCR-positive samples with CT of ⩽30 cycles), sequential multiplex PCR combined with fragment analysis and automated fluorescent capillary electrophoresis to differentiate serotypes [1, 2, 3, 4, 5, 6A/6B, 6C, 6,7C/(7B/40), 7F/7A, 9N/9L, 9V/9A, 10A, 10F/(10C/33C), 11A/11D, 12F/(12A/44/46), 13, 16F, 17F, 18/(18A/18B/18C/18F), 19A, 19F, 20(20A/20B), 21, 22F/22A), 23A, 23B, 24/(24A/24B/24F), 31, 34, 35A/(35C/42), 35B, 35F/47F, 38/25F and 39] was used [15].

Serotypes identified by real-time PCR as group level (6A/6C, 7F/7A, 9V/9A and 19F/19B/19C) were defined as PCV13 serotypes 6A, 7F, 9V and 19F, respectively.

PCR-positive samples that were negative for the serotypes included in the sequential multiplex PCR (including all vaccine serotypes) were classified as other non-vaccine serotypes (ONV).

Susceptibility to penicillin and cefotaxime was determined by the microdilution method according to the Clinical and Laboratory Standards Institute (CLSI) criteria. Current EUCAST breakpoints were used to interpret susceptibility [21]. A minimum inhibitory concentration (MIC) study was carried out at the National Microbiology Center. The penicillin breakpoints used were: MIC >0.06 mg/l, non-susceptible and MIC >2 mg/l, resistant. For cefotaxime, the breakpoint was: MIC >0.5 mg/l, non-susceptible.

### Statistical analysis

Proportions were compared using the *χ*^2^ test or Fisher's exact test, as appropriate. All statistical tests were two-tailed and statistical significance was established as *P* < 0.05. If the number of cases was zero, this was corrected by adding 0.5, and *P* values were calculated according to Sheskin's method [22]

Associations between variables were assessed using the odds ratio (OR) and 95% confidence intervals (CI).

The analyses were conducted using the Statistical Package for Social Sciences (SPSS 19.0 for Windows) and EPIDAT (program for the epidemiological analysis of tabulated data; version 3.1).

### Data confidentiality and ethical aspects

Informed consent was obtained from all individual participants included in the study. The study complies with the principles of the Declaration of Helsinki and the legal structure with respect to international human rights and biomedicine and protection of personal data laws. The Ethics Committee of Hospital Sant Joan de Déu approved the study.

## Results

### Baseline characteristics

During the study period, 263 cases of IPD were diagnosed in children aged ⩽17 years. Serotyping was not possible in ten cases (3.8%), which were excluded. Of the 253 cases included, 151 (59.7%) were male patients; 87 (34.4%) were children aged <2 years, 98 (38. 7%) in those aged 2–4 years, and 68 (26.9%) in those aged 5–17 years (Table 1).

**Table 1.** Demographic characteristics and clinical presentation in children aged ⩽17 years with IPD

	*N* (%)
	(253 cases)
*Age group*	
<2 years	87 (34.4)
2–4 years	98 (38.7)
5–17 years	68 (26.9)
*Sex*	
Male	151 (59.7)
*Seasonality*	
October–March	182 (72.0)
*Diagnosis*	
Culture only	62 (24.5)
PCR only	122 (48.2)
Culture + PCR	69 (27.3)
*Clinical presentation*	
Overall pneumonia	187 (73.9)
Complicated pneumonia	150 (80.2)
Occult bacteraemia	25 (9.9)
Meningitis	18 (7.1)
Other forms^a^	23 (9.1)
*ICU admission*	
Yes	49 (19.4)
*Underlying medical condition*	
Yes	15 (6.0)
*Case fatality rate*	2 (0.8)
Yes	
*Serotype groups*	
PCV13	163 (64.4)
Non-PCV13	90 (35.6)
*Pneumococcal vaccination*^b^	
PCV13	
⩾1 dose	82 (32.4)
Fully vaccinated	43 (17.0)
Partly vaccinated	39 (15.3)
No dose	170 (67.2)
Non-vaccinated (any PCV)	129 (51.2)
Unknown	1 (3.9)
*Antimicrobial susceptibility (n* *=* *122 strains)*	
Penicillin non-susceptible^c^	44 (36.1)
Cefotaxime non-susceptible	20 (16.4)

IPD, invasive pneumococcal disease; CFR, case fatality rate; ICU, intensive care unit; PCV13, 13-valent pneumococcal conjugate vaccine.

a Other forms: septic shock (7 cases), mastoiditis (7 cases), osteoarticular infection (6 cases), orbital cellulitis (2 cases), pancreatitis (1 case).

b 1 missing case.

c 4 cases were penicillin-resistant.

There was a seasonal variation, with 72% of cases detected during the cool months (October to March; *P* < 0.001).

With respect to PCV13 vaccination, 170 children (67.2%) were not immunised, 43 (17%) were fully immunised and 39 (15.4%) partly immunised; 129 children (51.0%) had received no dose of any conjugate vaccine. In one case, the vaccination status could not be determined.

Fifteen cases (6%) had a chronic disease, including congenital immunodeficiency (2 cases), congenital heart disease (3 cases), chronic pulmonary disease (1 case), chronic kidney disease (1 case), cerebrospinal fluid leak (1 case), or had received immunosuppressive therapy in the previous 6 months (7 cases).

**Fig. 1.**
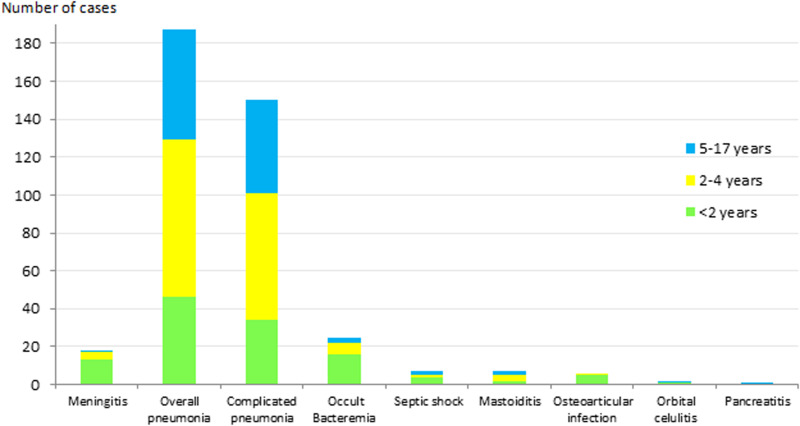
Distribution of IPD clinical presentation in children aged ⩽17 years by age group. IPD: invasive pneumococcal disease.

The most frequent clinical presentation was pneumonia (187 cases; 73.9%), of which 150 cases (80. 2%) were complicated pneumonia. This was followed by occult bacteraemia (25 cases; 9.9%) and meningitis (18 cases; 7.1%). The presentation in the remaining 23 cases (9.1%) was septic shock (7 cases; 2.8%), mastoiditis (7 cases; 2.8%), osteoarticular infection (6 cases; 2.3%), orbital cellulitis (2 cases; 0.8%) and pancreatitis (1 case; 0.4%). Figure 1 shows the distribution of clinical manifestations by the age group. Complicated pneumonia was the most frequent clinical presentation in all age groups.

Forty–nine cases (19.4%) were admitted to the ICU: 7 out of 7 (100%) with septic shock, 15 out of 18 (83.3%) with meningitis, 23 out of 150 (15.3%) with complicated pneumonia, 1 out of 7 (14.3%) with mastoiditis, 2 out of 37 (5.4%) with non-complicated pneumonia and 1 out of 25 (4%) with occult bacteraemia (ICU admission was caused by convulsions). Of the 49 ICU cases, 55.1% were caused by PCV13 serotypes, and 14.3% had an underlying disease.

Two patients died: an 18-month-old child with serotype-35F meningitis who had congenital immunodeficiency, and a 23-month-old child with septic shock caused by a non-vaccine serotype which was not identified.

### Serotypes and clinical presentation

**Fig. 2.**
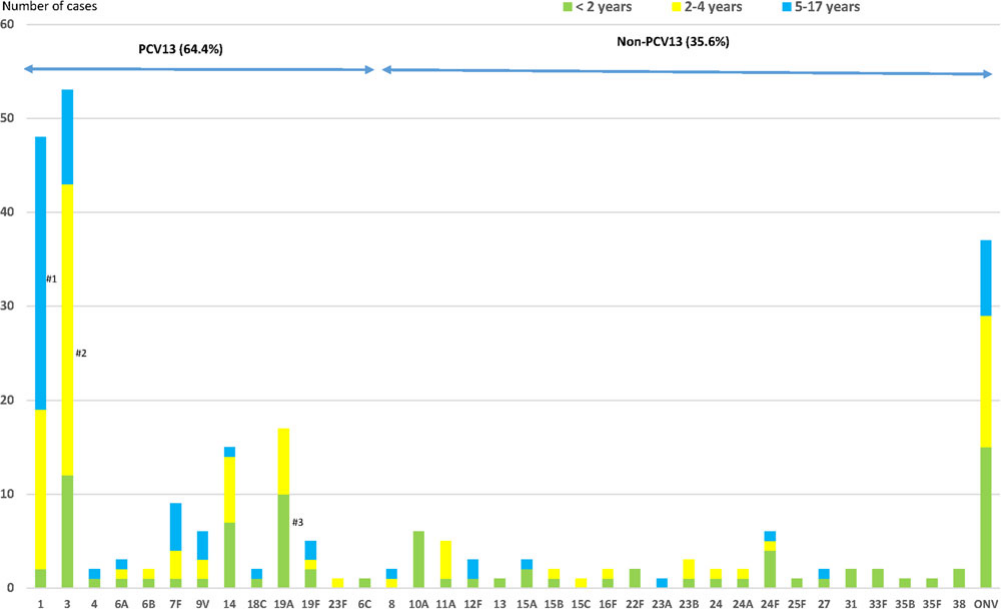
Distribution of *Streptococcus pneumoniae* serotypes causing IPD in children aged ⩽17 years by age groups. IPD: invasive pneumococcal disease. ONV: other non-vaccine serotypes. **#^1^**: *P* < 0.001; **#^2^**: *P* = 0.001; **#^3^**: *P* = 0.028.

One hundred and sixty-three (64.4%) cases were due to PCV13 serotypes (Fig. 2); 41.5% patients had received ⩾1 dose of PCV13 (34/82 cases) and 75.2% patients had not received any conjugated vaccine (97/129; *P* < 0.001).

The most frequent serotypes were: 3 (20.9%), 1 (19.0%), 19A (6.7%) and 14 (5.9%), all PCV13 serotypes.

Overall pneumonia and complicated pneumonia, respectively, were mainly caused by PCV13 serotypes (143/187 cases, 76.4% and 123/150 cases, 82.0%, respectively): 87.7% of IPD cases caused by PCV13 serotypes had pneumonia, compared with only 48.9% of IPD cases caused by non-PCV13 serotypes (OR 7.47, 95% CI 4.0–13.96). Similarly, 75.5% of cases caused by PCV13 serotypes led to complicated pneumonia compared with only 30% of IPD cases caused by non-PCV13 serotypes (OR 7.2, 95% CI 4.04–12.75) (Table 2). PCV13 serotypes were more likely to cause overall pneumonia and complicated pneumonia than non-PCV13 serotypes.

**Table 2.** Distribution of *Streptococcus pneumoniae* serotypes causing IPD in children aged ⩽17 years by clinical presentation

Serotype	Total		Meningitis			Overall pneumonia			Complicated pneumonia			Occult bacteraemia			Others		
	*n*	%	*n*	%	*P* value*	*n*	%	*P* value*	*n*	%	*P* value*	*n*	%	*P* value*	*n*	%	*P* value*
**PCV13**																	
**1**	48	19.0	0	0.0	–	47	97.9	<0.001	41	85.4	<0.001	0	0.0	–	1	2.1	ns
**3**	53	20.9	1	1.9	ns	49	92.5	0.001	44	83.0	<0.001	0	0.0	–	3	5.7	ns
**4**	2	0.8	0	0.0	–	1	50.0	ns	0	0.0	–	1	50.0	ns	0	0.0	–
**6A**^a^	3	1.2	0	0.0	–	3	100.0	ns	3	100.0	ns	0	0.0	–	0	0.0	–
**6B**	2	0.8	0	0.0	–	2	100.0	ns	1	50.0	ns	0	0.0	–	0	0.0	–
**7F**^a^	9	3.6	0	0.0	–	9	100.0	ns	9	100.0	ns	0	0.0	–	0	0.0	–
**9V**^a^	6	2.4	0	0.0	–	4	66.7	ns	4	66.7	ns	2	33.3	ns	0	0.0	–
**14**	15	5.9	1	6.7	ns	12	80.0	ns	9	60.0	ns	1	6.7	ns	1	6.7	ns
**18C**	2	0.8	0	0.0	–	0	0.0	–	0	0.0	–	2	100.0	0.009	0	0.0	–
**19A**	17	6.7	1	5.9	ns	15	88.2	ns	12	70.6	ns	0	0.0	–	1	5.9	ns
**19F**^a^	5	2.0	1	20.0	ns	1	20.0	ns	0	0.0	–	2	40.0	ns	1	20.0	ns
**23F**	1	0.4	0	0.0	–	0	0.0	–	0	0.0	–	1	100.0	ns	0	0.0	–
**Non-PCV13**																	
**6C**	1	0.4	0	0.0	–	1	100.0	ns	0	0.0	–	0	0.0	–	0	0.0	–
**8**	2	0.8	0	0.0	–	1	50.0		1	50.0	ns	0	0.0	–	1	50.0	ns
**10A**	6	2.4	1	16.7	ns	1	16.7	ns	0	0.0	–	3	50.0	0.014	1	16.7	ns
**11A**	5	2.0	0	0.0	–	3	60.0	ns	1	20.0	ns	0	0.0	–	2	40.0	ns
**12F**	3	1.2	0	0.0	–	1	33.3	ns	1	33.3	ns	2	66.7	0.026	0	0.0	–
**13**	1	0.4	1	100.0	ns	0	0.0	–	0	0.0	–	0	0.0	–	0	0.0	–
**15A**	3	1.2	1	33.3	ns	0	0.0	ns	0	0.0	–	1	33.3	ns	1	33.3	ns
**15B**	2	0.8	1	50.0	ns	0	0.0	–	0	0.0	–	1	50.0	ns	0	0.0	–
**15C**	1	0.4	0	0.0	–	0	0.0	–	0	0.0	–	1	100.0	ns	0	0.0	–
**16F**	2	0.8	1	50.0	ns	0	0.0	–	0	0.0	–	1	50.0	ns	0	0.0	–
**22F**	2	0.8	1	50.0	ns	0	0.0	–	0	0.0	–	0	0.0	–	1	50.0	ns
**23A**	1	0.4	0	0.0	–	0	0.0	–	0	0.0	–	0	0.0	–	1	100.0	ns
**23B**	3	1.2	0	0.0	–	1	33.3	ns	1	33.3	ns	1	33.3	ns	1	33.3	ns
**24**	2	0.8	0	0.0	–	1	50.0	ns	0	0.0	–	1	50.0	ns	0	0.0	–
**24A**	2	0.8	1	50.0	ns	1	50.0	ns	0	0.0	–	0	0.0	–	0	0.0	–
**24F**	6	2.4	1	16.7	ns	4	66.7	ns	2	33.3	ns	1	16.7	ns	0	0.0	
**25F**	1	0.4	0	0.0	–	0	0.0	–	0	0.0	–	1	100.0	ns	0	0.0	–
**27**	2	0.8	0	0.0	–	0	0.0	–	0	0.0	–	1	50.0	ns	1	50.0	ns
**31**	2	0.8	0	0.0	–	0	0.0	–	0	0.0	–	1	50.0	ns	1	50.0	ns
**33F**	2	0.8	0	0.0	–	1	50.0	ns	0	0.0	–	0	0.0	–	1	50.0	ns
**35B**	1	0.4	0	0.0	–	0	0.0	–	0	0.0	–	1	100.0	ns	0	0.0	–
**35F**	1	0.4	1	100.0	ns	0	0.0	–	0	0.0	–	0	0.0	–	0	0.0	–
**38**	2	0.8	0	0.0	–	1	50.0	ns	1	50.0	ns	0	0.0	–	1	50.0	ns
**ONV**	37	14.6	5	13.5	–	28	75.7	–	20	54.1	–	0	0.0	–	4	10.8	–
**PCV13**	163	64.4	4	2.5		143	87.7	<0.001	123	75.5	<0.001	9	5.5		7	4.3	
**Non-PCV13**	90	35.6	14	15.6	<0.001	44	48.9		27	30.0		16	17.8	0.002	16	17.8	<0.001
**Total**	253	100.0	18	7.1	–	187	73.9	–	150	59.3	–	25	9.9	–	23	9.1	–

IPD, invasive pneumococcal disease; ONV, other non-vaccine serotypes.

* The *P* value indicates differences in the relationship between a serotype and a clinical form with respect to the other serotypes. ‘ns’ indicates *P* value⩾0.05.

a **7F: 4 out of 9** cases were identified as group level (7F/7A); **9V**: **2 out of 6** cases were identified as group level (9V/9A); 19F: **1 out of 5** case was identified as group level (19F/19B/19C); 6A: **2 out of 3** cases were identified as group level (6A/6C).

A total of 92.5% of IPD cases caused by serotype 3 were overall pneumonia and 83% of IPD cases caused by serotype 3 were complicated pneumonia. Serotype 3 caused overall pneumonia and complicated pneumonia more frequently than the other clinical forms (OR 5.5, 95% CI 1.9–15.92 and OR 4.3, 95% CI 2.01–9.35, respectively).

Serotype 1 was also associated with overall pneumonia (OR 21.82, 95% CI 2.95–161.63) and complicated pneumonia (OR 5.16, 95% CI 2.21–12.04). Serotype 1 was more likely to cause overall pneumonia and complicated pneumonia than other clinical forms (OR 21.82, 95% CI 2.95–161.63 and OR 5.16, 95% CI 2.21–12.04, respectively).

Meningitis was mainly caused by non-PCV13 serotypes (14/18, 77.8%). Although there was no predominant serotype, 15.6% of IPD cases caused by non-PCV13 serotypes presented as meningitis, compared with only 2.5% of cases caused by PCV13 serotypes (OR 7.32, 95% CI 2.33–22.99).

Occult bacteraemia was also caused mainly by non-PCV13 serotypes (16/25, 64%), and 17.8% of IPD cases caused by non-PCV13 serotypes presented as occult bacteraemia compared with only 5.5% of cases caused by PCV13 serotypes (OR 3.6, 95% CI 1.56–8.76). Some non-PCV13 serotypes were associated with occult bacteraemia, namely 12F (OR 19.73, 95% CI 1.72–226.12) and 10A (OR 10.22, 95% CI 1.94–53.74). Serotype 18C, a PCV13 serotype was also associated with occult bacteraemia (OR 48.61, 95% CI 2.26–1043.02), but because there are only two cases with this serotype the finding cannot be evaluated.

Other clinical presentations were caused mainly by non-PCV13 serotypes (16/23; 69.6%), with 17.8% of IPD cases caused by non-PCV13 serotypes presenting as other clinical presentations compared with only 4.3% of IPD cases caused by PCV13 serotypes (OR 4.81, 95% CI 1.90–12.22).

### Serotypes and clinical presentation according to age group

PCV13 serotypes were those most frequently detected in 5–17-year-olds, representing 77.9% of all serotypes in this age group (OR 2.41, 95% CI 1.27–4.59) and non-PCV13 serotypes were the most frequently detected serotypes (55.2%) in the <2 years age group (OR 3.63, 95% CI 2.10–6.29). In the 5–17 years age group, IPD was more likely to be caused by PCV13 serotypes.

Serotype 19A was more frequent in the <2 years age group, serotype 3 in the 2–4 years age group (58.5%; OR 2.79, 95% CI 1.50–5.20) and serotype 1 in the 5–17 years age group (60.4%; OR 6.50, 95% CI 3.31–12.77) (Fig. 2). Serotype 19A more frequently caused IPD in the <2 years age group (58.8%; OR 2.95, 95% CI 1.08–8.05), as did serotype 3 in the 2–4 years age group (58.5%; OR 2.79, 95% CI 1.50–5.20) and serotype 1 in the 5–17-years age group (60.4%; OR 6.50, 95% CI 3.31–12.77) (Fig. 2).

**Fig. 3.**
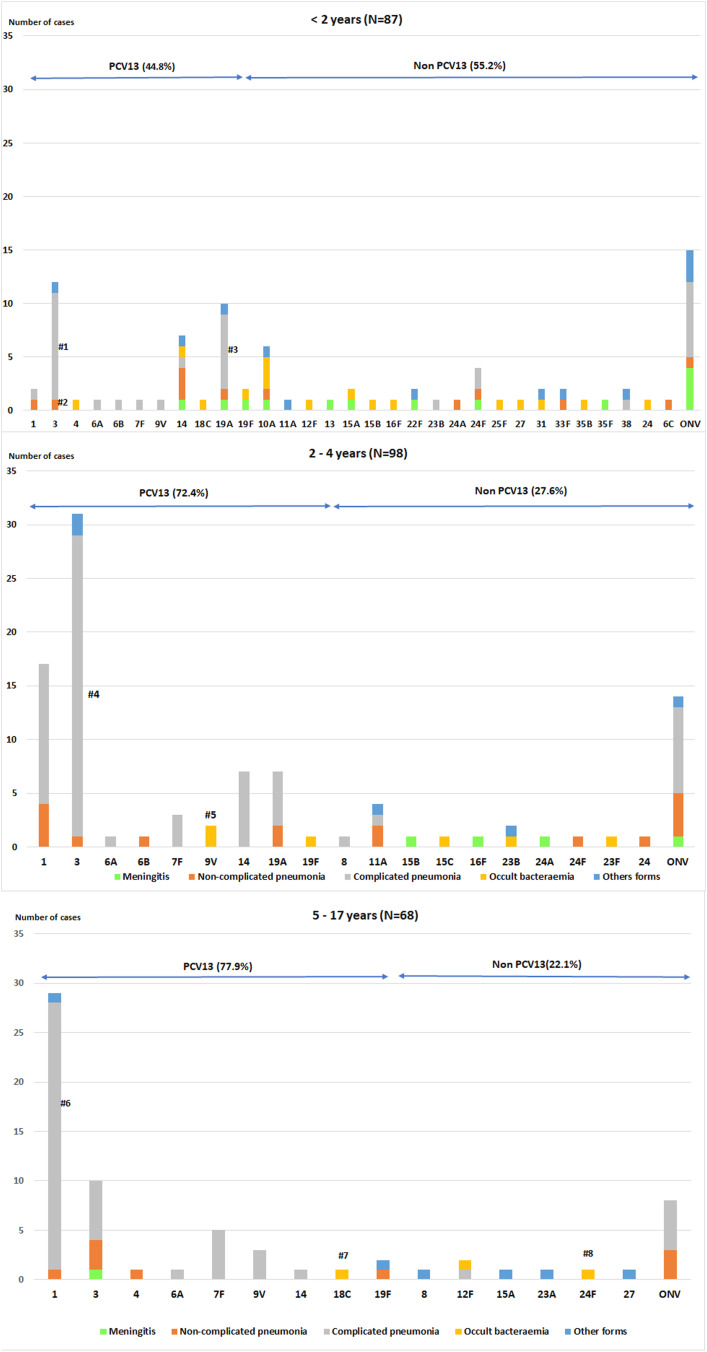
Distribution of *Streptococcus pneumoniae* serotypes in children aged ⩽17 years by clinical presentation.
**<2 age group**. **#^1^**: *P* < 0.001; **#^2^:** *P* = 0.004 (non-complicated and complicated pneumonia); **#^3^:***P* = 0.033.**2–4 age group**. **#^4^**: *P* = 0.002; **#^5^**:*P* = 0.003**5–17 age group**. **#^6^**:*P* < 0.001; **#^7^**: *P* = 0.004; **#^8^**: *P* = 0.004 The *P* values indicate the differences in the relationship between a serotype and a clinical form with respect to the other serotypes. IPD: invasive pneumococcal disease. ONV: other non-vaccine serotypes.

Table 3 and Figure 3 show the serotype distribution by age groups and the association to clinical presentation.

**Table 3.** Distribution of *Streptococcus pneumoniae* serotype groups causing IPD in children aged ⩽17 years by clinical presentation and age group

Age groups	Serotype group	Total					Clinical presentation											
				Meningitis			Overall pneumonia			Complicated pneumonia			Occult bacteraemia			Other forms		
		*n*	%	*n*	%	*P* value*	*n*	%	*P* value*	*n*	%	*P* value*	*n*	%	*P* value*	*n*	%	*P* value*
**0–17 years**																		
	PCV13	163	64.4	4	2.5	–	143	87.7	<0.001	123	75.5	<0.001	9	5.5	–	7	4.3	–
	Non-PCV13	90	35.6	14	15.6	<0.001	44	48.9	–	27	30.0	–	16	17.8	0.002	16	17.8	<0.001
	**Total**	253	100.0	18	7.1		187	73.9		150	59.3		25	9.9		23	9.1	
**<2 years**																		
	PCV13	39	44.8	3	7.7	–	29	74.4	<0.001	23	59.0	0.001	4	10.3	–	3	7.7	–
	Non-PCV13	48	55.2	10	20.8	ns	17	35.4	–	11	22.9	–	12	25	ns	9	18.8	ns
	**Total**	87	100.0	13	14.9		46	52.9		34	39.1		16	18.4		12	13.8	
**2–4 years**																		
	PCV13	71	72.4	0	0	–	65	91.5	0.004	57	80.3	<0.001	4	5.6	–	2	2.8	–
	Non-PCV13	27	27.6	4	14.8	0.005	18	66.7	–	10	37.0	–	2	7.4	ns	3	11.1	ns
	**Total**	98	100.0	4	4.1		83	84.7		67	68.4		6	6.1		5	5.1	
**5–17 years**																		
	PCV13	53	77.9	1	1.9	ns	49	92.5	0.006	43	81.1	0.002	1	1.9	–	2	3.8	–
	Non-PCV13	15	22.1	0	0	–	9	60.0	–	6	40.0	–	2	13.3	ns	4	26.7	0.018
	**Total**	68	100.0	1	1.5		58	85.3		49	72.1		3	4.4		6	8.8	

* The *P* value indicates differences in the relationship between a serotype group and a clinical form with respect to the other serotypes.

‘ns’ indicates *P* value⩾0.05.

In children aged <2 years, PCV13 serotypes were associated with overall pneumonia (OR 5.29, 95% CI 2.08–13.41) and complicated pneumonia (OR 4.84, 95% CI 1.91–12.22). Serotype 3 caused overall pneumonia in 91.7% of cases (OR 12.57, 95% CI 1.54–102.33) and complicated pneumonia in 83.3% (OR 10.62, 95% CI 2.16–52.3). Serotype 19A was associated with complicated pneumonia (OR 4.32, 95% CI 1.03–18.07) in 70% of cases.

In the 2–4 years age group, PCV13 serotypes were associated with overall pneumonia (OR 5.42, 95% CI 1.70–17.23) and complicated pneumonia (OR 6.92, 95% CI 2.61–18.36), while non-PCV13 serotypes were associated with meningitis (OR 27.38, 95% CI 1.42–527.7). Serotype 3 was associated with complicated pneumonia (OR 6.7, 95% CI 1.85–24.24) and serotype 9V with occult bacteraemia (OR 102.7, 95% CI 4.27–2472.3).

In the 5–17 years age group, PCV13 serotypes were associated with overall pneumonia (OR 8.17, 95% CI 1.91–34.86) and complicated pneumonia (OR 6.05, 95% CI 1.81–20.18), whereas non-PCV13 serotypes were associated with other clinical forms (OR 9.3, 95% CI1.51–57.12). Serotype 1 was associated with complicated pneumonia (OR 10.43, 95% CI 2.17–50.12).

### Serotypes and method of diagnosis

The diagnosis was made by culture in 62 cases (24.5%), culture and real-time PCR in 69 (27.3%) and real-time PCR in 122 (48.2%).

PCV13 and non-PCV13 serotypes showed no significant difference in the percentage of cases diagnosed only by PCR (52.1% *vs*. 41.1%).

There were significant differences between serotypes in the method of diagnosis: 86.8% of cases due to serotype 3 were diagnosed only by real-time PCR *vs.* 38% of cases due to other serotypes (*P* < 0.001). By contrast, other serotypes, such as serotype 14 and 24F, were diagnosed less frequently by real-time PCR (13.3%, 2/15 and 0%, 0/6) than the other serotypes (*P* = 0.005 and *P* = 0.030, respectively).

### Non-susceptibility to antibiotics by serotypes

Antimicrobial susceptibility was studied in 122 (93.1%) strains isolated. Forty-four cases (36.1%) were non-susceptible to penicillin (MIC > 0.06), and four cases (3.3%) were penicillin-resistant. Twenty cases (16.4%) were also non-susceptible to cefotaxime (Table 4).

**Table 4.** Distribution of *Streptococcus pneumoniae* serotypes causing IPD in children aged ⩽17 years by non-susceptibility to antibiotics

Serotype	Total cases	Cases analysed		Penicillin >0.06 ^a^			Penicillin>2^b^			Cefotaxime>0.5^c^			Penicillin >0.06 + Cefotaxime>0.5^d^		
	*n*	*n*	%	*n*	%	*P* value*	*n*	%	*P* value*	*n*	%	*P* value*	*n*	%	*P* value *
**PCV13**															
**1**	48	27	56.3	0	0.0	–	0	0.0	–	0	0.0	–	0	0.0	–
**3**	53	6	11.3	0	0.0	–	0	0.0	–	0	0.0	–	0	0.0	–
**4**	2	2	100.0	0	0.0	–	0	0.0	–	0	0.0	–	0	0.0	–
**6A**^e^	3	1	33.3	0	0.0	–	0	0.0	–	0	0.0	–	0	0.0	–
**6B**	2	1	50.0	1	100	ns	0	0.0	–	0	0.0	–	0	0.0	–
**7F**^e^	9	1	11.1	0	0.0	–	0	0.0	–	0	0.0	–	0	0.0	–
**9V**^e^	6	5	83.3	1	20.0	ns	0	0.0	–	1	20.0	ns	1	20.0	ns
**14**	15	11	73.3	11	100.0	<0.001	0	0.0	–	9	81.8	<0.001	9	81.8	<0.001
**18C**	2	2	100.0	0	0.0	–	0	0.0	–	0	0.0	–	0	0.0	–
**19A**	17	11	64.7	10	90.9	<0.001	3	27.3	0.002	6	54.5	0.003	6	54.5	0.003
**19F**^e^	5	4	80.0	3	75.0	ns	1	25.0	ns	1	25.0	ns	1	25.0	ns
**23F**	1	1	100.0	0	0.0	–	0	0.0	–	0	0.0	–	0	0.0	–
**Non-PCV13**															
**6C**	1	1	100.0	0	0.0	–	0	0.0	–	0	0.0	–	0	0.0	–
**8**	2	2	100.0	0	0.0	–	0	0.0	–	0	0.0	–	0	0.0	–
**10A**	6	5	83.3	0	0.0	–	0	0.0	–	0	0.0		0	0.0	
**24F**	6	6	100.0	5	83.3	0.023	0	0.0	–	0	0.0	–	0	0.0	–
**11A**	5	5	100.0	3	60.0	ns	0	0.0	–	3	60.0	0.031	3	60.0	0.031
**12F**	3	3	100.0	0	0.0	–	0	0.0	–	0	0.0	–	0	0.0	–
**13**	1	1	100.0	0	0.0	–	0	0.0	–	0	0.0	–	0	0.0	–
**15A**	3	3	100.0	1	33.3	ns	0	0.0	–	0	0.0	–	0	0.0	–
**15B**	2	2	100.0	0	0.0	–	0	0.0	–	0	0.0	–	0	0.0	–
**15C**	1	1	100.0	0	0.0	–	0	0.0	–	0	0.0	–	0	0.0	–
**16F**	2	2	100.0	1	50.0	ns	0	0.0	–	0	0.0	–	0	0.0	–
**22F**	2	2	100.0	0	0.0	–	0	0.0	–	0	0.0	–	0	0.0	–
**23B**	3	3	100.0	3	100.0	0.045	0	0.0	–	0	0.0	–	0	0.0	–
**24**	2	2	100.0	2	100.0	ns	0	0.0	–	0	0.0	–	0	0.0	–
**24A**	2	2	100.0	2	100.0	ns	0	0.0	–	0	0.0	–	0	0.0	–
**27**	2	2	100.0	1	50.0	ns	0	0.0	–	0	0.0	–	0	0.0	–
**31**	2	2	100.0	0	0.0	–	0	0.0	–	0	0.0	–	0	0.0	–
**33F**	2	2	100.0	0	0.0	–	0	0.0	–	0	0.0	–	0	0.0	–
**35B**	1	1	100.0	0	0.0	–	0	0.0	–	0	0.0	–	0	0.0	–
**35F**	1	1	100.0	0	0.0	–	0	0.0	–	0	0.0	–	0	0.0	–
**38**	2	2	100.0	0	0.0	–	0	0.0	–	0	0.0	–	0	0.0	–
**PCV13**	163	72	44.2	26	36.1	ns	4	5.6	ns	17	23.6	0.010	17	23.6	0.010
**Non-PCV13**	90	50	55.6	18	36.0		0	0.0		3	6.0		3	6.0	
**Total**	253	122	48.2	44	36.1	–	4	3.3	–	20	16.4	–	20	16.4	–

IPD, invasive pneumococcal disease; ONV, other non-vaccine serotypes.

a Number and percentage of isolates of a given serotype, with penicillin MIC >0.06 mg/l.

b With penicillin MIC >2.0 mg/l.

c With cefotaxime MIC >0.5 mg/l.

d Not susceptible to either penicillin or cefotaxime.

e **7F: 4 out of 9** cases were identified as group level (7F/7A); **9V**: **2 out of 6** cases were identified as group level (9V/9A); **19F**: **1 out of 5** cases was identified as group level (19F/19B/19C); **6A**: **2 out of 3** cases were identified as group level (6A/6C).

* The *P* value indicates differences in the relationship between a serotype and non-susceptibility to an antibiotic with respect to the other serotypes. ‘ns’ indicates *P* value⩾0.05.

With respect to the clinical form, 5 out of 44 cases with MIC > 0.06 were considered resistant due to the meningeal location (serotypes 14, 19A, 19F, 24A and 24F). One case (serotype 14) was also non-susceptible to cefotaxime.

There were no significant differences in the percentage of non-susceptible penicillin strains between PCV13 (36.1%) and non-PCV13 serotypes (36.0%). PCV13 serotypes were associated with strains not susceptible to cefotaxime (*P* = 0.010) and with isolates not susceptible to either penicillin or cefotaxime (*P* = 0.010).

Serotype 19A showed a penicillin MIC of >0.06 mg/l in 90.9% of cases studied (*P* < 0.001) and MIC >2 mg/l in 27.3% (*P* = 0.002). In addition, 54.5% of serotype 19A strains were non-susceptible to cefotaxime (*P* = 0.003) and non-susceptible to penicillin (*P* = 0.003).

All serotype 14 strains (100%) showed a penicillin MIC of >0.06 mg/l (*P* < 0.001) and 81.8% of cases were non-susceptible to both cefotaxime (*P* < 0.001) and penicillin (*P* < 0.001).

Serotype 11A showed non-susceptibility to cefotaxime in 60% of strains compared with 14.5% for other serotypes (*P* = 0.031). These strains were also non-susceptible to penicillin (*P* = 0.031).

Other serotypes showed significant differences in antimicrobial susceptibility, namely 24F, which showed a penicillin MIC of >0.06 mg/l in 83.3% (*P* = 0.023), and 23B in 100% (*P* = 0.045).

## Discussion

PCV13 serotypes caused 64.4% of IPD cases in children aged ⩽17 years during the study period, and the most frequent serotypes were 3, 1, 19A and 14. Other studies in Catalonia have shown a higher frequency of PCV13 serotypes, despite the decreased incidence in all age groups in this period [23]. However, other authors have found a higher percentage of non-PCV13 serotypes. Makwana *et al*. observed that 77.5% of cases of IPD in children aged <5 years in England and Wales between 2010 and 2016 were caused by non-PCV13 serotypes although there was a high immunisation rate with 97.3% having received ⩾1PCV13 dose [24]. A study conducted in Madrid (Spain) between 2012 and 2015, with vaccination coverages between 67% and 95%, found that non-PCV13 serotypes accounted for 68.2% of cases in children aged <15 years [25], somewhat higher than the 55.2% described by Janoir *et al*. [14] in France in 2011–2012 in the same age group, with a vaccination coverage of at least 90%. The differences between these results might be explained, at least in part, by a lower PCV13 coverage (about 64% in children aged <2 years) in Catalonia than in other areas because during the study period, PCV13 was not included in the Catalan immunisation schedule [12]. In fact, a systematic review by Balsells *et al*. in children in the post-PCV era found large differences between countries. Non-PCV13 serotypes accounted for 42.2% of childhood IPD cases, but marked regional differences were observed, from 9.2% in one Eastern Mediterranean country to 71.9% in Europe [26].

With respect to the distribution of the main serotypes, Ceyhan *et al*. [27] found that, in Turkish children aged <18 years with 25.5% of IPD cases having received any PCV dose, serotype 19F was the most frequent, followed by 14, 3 and 6B (in equal amounts), while Camilli *et al*. [28], in Italian children aged <5 years in 2012–2014 with PCV coverage at 24 months of age of about 80% found that serotypes 1, 19A and 14 were among the four most frequent serotypes, together with 24F. In addition to the differences in PCV coverage, the diagnostic techniques used in these studies might help to explain the differences observed [29]. In our study, serotype 3 was the most frequent and also the most frequently diagnosed only by real-time PCR (87% of cases due to serotype 3 were diagnosed only by real-time PCR). When real-time PCR is not available for the diagnosis, another distribution of serotypes in the same age groups may be found, because some serotypes are more antimicrobial-resistant than others and real-time PCR makes it possible to diagnose the more sensitive serotypes. Obando *et al*. [30] found evidence of pneumococcal infection in 84% of culture-negative pleural fluid samples on the basis of *ply* or *wzg* gene detection by PCR; these cases were significantly more likely to have received antimicrobial drug therapy before sampling. In our study 48% of IPD cases were diagnosed only by real-time PCR, which served to increase the etiologic diagnosis and serotype identification.

Serotype 19A was associated with age <2 years, serotype 3 with the 2–4 years age group and serotype 1 with the 5–17 years age group. Other studies have found similar results, with 100% of cases due to serotype 19A in children aged <2 years, 63.6% of cases due to serotype 3 in the 2–4 years age group and 80% of cases due to serotype 1 in children aged >4 years [21].

Compared with the pre-PCV13 era, [23] in our region the most frequent serotype was 19A (24.6%) in children aged <2 years and serotype 1 (46.8%) in children aged 2–4 years, while serotype 3, now the most frequent, represented only 2.8% and 4.2% of cases in these age groups, respectively. This may be explained by the replacement of serotypes.

PCV13 serotypes were associated with overall pneumonia and complicated pneumonia in children aged ⩽17 years, mainly serotype 19A in children aged <2 years, serotype 3 in those aged <5 years and serotype 1 in the 5–17 years age group. A Polish study conducted between 2011 and 2013 in all age groups, found that PCV13 serotypes caused 78.4% of cases of pneumonia, and serotype 3 and serotype 1 were associated with pneumonia, as in our study [31].

In our study, non-PCV13 serotypes were associated with occult bacteraemia, especially 10A and 12F, and with meningitis in the 2–4 years age group. Similarly, other authors have reported that 90% of meningitis cases and 64% of occult bacteraemia cases during 2011–2012 were produced by non-PCV13 serotypes [32].

The percentage of strains not susceptible to penicillin (36.1%) or to cefotaxime (16.4%) was higher than that described by other authors [23, 25]. Serotypes 19A and 14 were associated with strains not susceptible to penicillin or cefotaxime, whereas serotype 23B and 24F were not susceptible to penicillin. Other authors have also described non-susceptibility to penicillin in serotypes 19A and 14 [28, 31], serotype 24F [14, 28] and serotype 23B [33]. Skoczyńska *et al*. [31] found that non-susceptibility to penicillin was also associated with vaccine-serotypes 19F, 6B and 9V, which in our study could not be demonstrated, perhaps due to the small number of strains studied. They found an association between non-susceptibility to cefotaxime in serotype 19A and 14, as in our study. The non-susceptibility to antibiotics of non-PCV13 serotypes is concerning and should be monitored.

Among the strengths of the study is the high percentage of cases serotyped, since serotyping was not possible in only 3.8% of cases. The same diagnostic techniques (culture and real-time PCR) were used uniformly in all cases, improving the diagnostic sensitivity and the range of serotypes identified, which permitted a more precise estimate of the burden of IPD.

There were some limitations. First, the population studied corresponds to three paediatric hospitals, although these hospitals serve 32% of the population aged ⩽17 years requiring hospitalisation in Catalonia. In the subgroup analyses, the small number of cases observed in some situations may explain why the confidence intervals of the measure of association were very wide. Finally, antibiotic sensitivity could only be studied in 48% of cases serotyped, since a large part of the cases were diagnosed by real-time PCR.

## Conclusions

PCV13 serotypes were the most frequently found IPD serotypes in children aged ⩽17 years, especially serotype 19A in children aged <2 years, serotype 3 in the 2–4 years age group and serotype 1 in the 5–17 years age group.

Non-PCV13 serotypes were the main cause of meningitis, occult bacteraemia and other clinical presentations, while PCV13 serotypes were mainly responsible for pneumonia.

PCV13 and non-PCV13 cases presented a high frequency of non-susceptibility to penicillin. Non-susceptibility to both penicillin and cefotaxime was associated with serotypes 19A and 14, and serotype 19A was associated with resistance to penicillin.

The non-susceptibility to antibiotics of non-PCV13 serotypes is concerning and should be monitored to apply the appropriate disease prevention strategies.

## Data Availability

No additional data are available.
